# Bibliographical Notices

**Published:** 1855-07

**Authors:** 


### BIBLIOGRAPHICAL NOTICES.

*Surgical Reports and Miscellaneous Papers on Medical Subjects.* By George Hayward, M.D., President of the Massachusetts Medical Society; Fellow of the American Academy of the Arts and Sciences; late Professor of Surgery in Harvard University, and Consulting Surgeon to the Massachusetts General Hospital. Boston: Phillips, Sampson & Co. 1855. 12mo., pp. 452, including index.

We doubt not that the "Reports" and "Papers" embodied in the above neat and unpretending volume will meet with a general and hearty welcome. Our readers will recognize the most of them as old and valued acquaintances, well known to the profession through the pages of the American Journal of the Medical Sciences, and other sources, in which they first appeared. Their previous reputation would secure to them a favorable reception which the present improved and convenient form will render doubly certain; but the high standing of their veteran and able author as one of the first of the surgeons of New England, clear-headed, learned and accomplished in practice and teaching, must add unusual interest to these mementoes of his long and very active career. He has earned the thanks of his brethren in carefully arranging and extending his various essays so as to bring them permanently within the reach of every one; and we hope that the example which, in common with his former colleague Dr. Bigelow, he has thus presented, will lead to similar efforts on the part of some of his associates, as well as others equally esteemed and distinguished in different parts of our country.

Dr. Hayward's contributions, as he reminds us, are the results of more than forty years' experience, and are offered in a collected form "from a belief that some of them would be useful from the facts and tables they contain, and in the hope that all might be read with advantage by students and the younger members of the profession." He further very modestly suggests that they might "be occasionally consulted by those somewhat advanced in practice, whose time was too much occupied to allow an examination of more extended works on the subjects of which they treat." The original importance of several of them is materially enhanced, not only by the revision to which all have been subjected, but by the addition of new cases, statistics and other matter, which give them the aspect and freshness of entirely new articles.

We find in the first eighty pages a useful series of clinical reports and considerations on surgery and operations in the Massachusetts General Hospital. Many practical hints of value may be derived from the study of the cases touched upon in these reports, while still more may be gathered from the discussion of certain mooted questions, which are presented here and there with considerable effect. Some of these latter are sufficiently recent and important to justify a special comment were the space allowed us; but we are obliged to pass at once to the next subject of examination. This is presented in a "Report on the Permanent Cure of Reducible Hernia," submitted to the American Medical Association at Richmond, in 1852. The conclusions of this paper may be regarded as in accordance with the views of the great body of the profession on both sides of the Atlantic. They agree so fully with the doctrine which we have repeatedly maintained, that we are tempted to extract them here:

"1. That there is no surgical operation at present known which can be relied on, with confidence, to produce, in all instances, or even in a large proportion of cases, a radical cure of hernia."

"2. That they regard the operation of injection by the subcutaneous method as the safest and the best. This will probably, in some cases, produce a radical cure, and, in many others, will afford great relief."

"3. That compression, when properly employed, is, in the present state of our knowledge, the most likely means of effecting a radical cure in the greatest number of cases." pp. 116—117.

We have met with so many undoubted and unequivocal cures of hernia, produced by the action of a truss alone, that we are satisfied of the entire truth and force of the third conclusion.

Next we have several chapters in succession occupied with different subjects, some of them interesting to the general reader, and all attractive to the professional student. Among them are the well known "Statistics of Amputations," now brought up to 1850; "Cases of Vesico-Vaginal Fistula," of which eight cases, with the accompanying and concluding remarks, are new, and of recent date; a chapter on anæsthetic agents, in which we rejoice to learn that he continues his preference for sulphuric ether and his positive objection to chloroform, chloric ether and other similar mixtures; a chapter on the "Statistics of Consumption;" one on "Legalizing Anatomy;" another on the "Contagion of Cholera;" a "Case of Hydrophobia;" a "Case of Paruria Inops;" also chapters on other subjects which we need not name, but which we would heartily commend to the perusal of our readers, for their especial gratification as well as practical improvement.

It would afford us sincere pleasure, had we room for it, to dwell upon many portions which have struck us as particularly deserving of attention in these instructive essays. We leave them with regret, on account of their varied intrinsic merits; but still more, we confess, because of our personal regard and respect for their worthy author. He is one whose teachings and example we are hardly yet prepared to look upon as among the lights that are destined henceforth to shine only in the past. We earnestly trust that there is yet awaiting him a brighter and busier future in the highest labors of his calling; and that we may all live to enjoy the fruits of his unabated industry in the further record of studies and experience which, at the close of half a century, are still in the vigor of their years.

*A Practical Treatise on the Diseases Peculiar to Women; Illustrated by Cases derived from Hospital and Private Practice. Third American, from the Third London Edition.* By SAMUEL ASHWELL, Obstetric Physician and Lecturer to Guy's Hospital, London. Blanchard & Lea.

Dr. Ashwell, in the Preface to his first edition, informs us that for the last twenty years his attention has been particularly directed to the important branch of medical science treated of in this excellent work; during a great part of which period Guy's Hospital, with its extensive lying-in Charity, and a large private practice in female diseases, have afforded him, as he himself candidly admits, "opportunities falling to the lot of few practitioners."

At the same time that he modestly disclaims any exclusive or undue deference on that account, for the opinions expressed and the practice recommended in the work, the author trusts that the conscientious watchfulness with which he has endeavored to use the opportunities and materials so abundantly bestowed on him, will secure for his labors the approval and confidence of his professional contemporaries.

In truth, the ability of this work must command the respect of the whole profession. Its unaffected simplicity, descriptive clearness, and sound practical doctrines, without a tinge of hobbyism, (the rarest virtue in our modern medical literature,) place it, as a practical treatise on female diseases, far in advance of any other with which we are acquainted in the English language.

To a reviewer, watching for an opportunity to display his critical acumen, Dr. Ashwell's perfect knowledge of his subject, backed by his sound discriminating sense and unaffected style, is somewhat baffling. He may differ with his author on some always debateable points which never have been and probably never will be settled, but to find fault would be hypercritical and even presumptuous.

Thus impressed, we shall endeavor, as far as our limited space permits, to give our readers some idea of the merits of his book, although we feel that we cannot, by our necessarily cursory analysis, convey more than a very faint conception of its importance and usefulness.

Dr. Ashwell arranges the diseases of women in two divisions: in the first he places the functional, and in the second the organic sexual diseases.

The functional diseases treated of are, chlorosis, amenorrhœa, dismenorrhœa, menorrhagia, leucorrhœa, and hysteria with irritable uterus. These subjects of course include the different complications, together with the disorders attendant on the natural cessation of menstruation; each disease and each complication is illustrated by cases, minutely described, together with the detail of treatment and the results; where death occurred, the post-mortem investigations are carefully and clearly given. In addition to all this, it may be mentioned that a useful collection of formulæ of all the remedies employed is included; thus giving to the younger members of the profession what they most require, viz: the minutiæ of the practice as well as the general experience of an able and conscientious physician, who has seen and studied by the bedside what he describes, and practices what he teaches.

It is somewhat trite to repeat the established truth, that a good practitioner cannot be made by books alone, but we are satisfied that all that any one man can do, towards instructing and guiding his less experienced, less gifted, or less fortunate professional brethren in the diagnosis and treatment of female diseases, has been accomplished by Dr. Ashwell in the treatise before us.

Our author's definition of chlorosis is as follows:

"A peculiar affection of the general health, in which debility, languor, and deranged stomachic functions are prominent symptoms; most frequently occurring when puberty is, or ought to be established, although it may exist at any subsequent period; always characterized by anæmia of the system, and a yellowish, dirty green pallor of the surface. When a disease of early youth, almost invariably connected either with entire absence of menstruation, or with a scanty, painful, and irregular performance of the function; and if a disease of later life, in addition to these causes it may have been preceded and produced by menorrhagia or leucorrhœa."

Chlorosis is properly divided into mild or incipient, inveterate or confirmed, and a complicated disease.

The mild form is clearly and graphically described; such a patient, says Dr. A.,

"Is languid, soon fatigued, and therefore inactive; she is not cheerful, but dull and listless; sometimes perverse and sullen, and prone to solitude. Her appetite is capricious; it either fails altogether or she craves for unwholesome food. Her complexion is altered; though always pale, it is now much more so; the bowels are constipated; the tongue is of a dirty paste white; the breath is offensive; she suffers from flatulence; the slightest exertion fatigues and induces short breathing; frequent, severe and peculiar headaches; palpitation of the heart and pain in the side are common occurrences; the pulse is quick, weak and compressible, and sometimes fluttering. The catamenia, if not absolutely delayed, have scarcely appeared, the discharge being pale in color and scanty in quantity."

Confirmed or inveterate chlorosis is of course but an aggravation of the same series and conditions, with other violent and alarming sympathetic affections, such as hysteria, with cerebral, pulmonary or abdominal difficulties, so that a careless or uninstructed observer might be blinded to the original affection, and suppose that his business consisted in combating an organic disease of the brain, lungs, liver or heart.

All the complications which the author's opportunities have enabled him to observe in chlorotic patients are described and illustrated by cases.

We give the following interesting case of cerebral complication as a specimen of the thorough and careful manner in which Dr. A. places the practical part of the work before his readers:

"Emily ———, aged 17, a tall, thin girl, of florid complexion and of intelligent appearance, was admitted Feb. 5th, 1836. She has always been weakly, and for the last four or five years, has been subject to chest affections, from which she has been free since the existence of her present malady. Two years ago she had phrenitis, and has since been in imperfect health, being often seized with aggravated fits of hysteria, so that she falls and remains insensible and motionless for hours together. She is now deaf, and has since had otorrhœa, but its presence was attributable to an accidental injury of the meatus externus. She has intense headache, chiefly affecting the occiput. The cephalalgia was unusually severe in December 1835, and soon afterwards her right foot and hand were frequently in agitation. A month subsequently she lost all control over them, but since this time there has been no aggravation of the pain. Ten days ago she was delirious, and remained so for a few hours. Menstruation has been only once regular and natural. Her present symptoms are,—dulness, almost imbecility of intellect; constant and rather acute occipital pain; frequent but not very violent agitation of the right side, with occasional spasms of the left; little or no affection of the face; no difficulty of articulation. Bowels open by purgative medicine; skin soft and moist.

℞. Pulv. scam. c. cal. grs. xv. statim.

℞. Ferri sub-carb. ʒj, quartâ quâque horâ.

To take half a pint of porter daily, and use the flesh brush.

This treatment was pursued for a fortnight with advantage, and the daily reports exhibit progressive amendment; the agitation is decreased and the pain in the head diminished.

℞. Zinci sulph. grs. ij. ter die. Balneum pluviale omni aurora.

Feb. 22d. The agitation is much less, and she has recovered considerable power of the left hand, but not so much of the leg. Bowels open; tongue clean.

25th. The agitation has somewhat increased, and she complains of pain in the affected arm and leg.

Augeatur dosis zinci. sulph. ad gr. iv. ter die.

She continued to improve until March 11, when the nurse reported that she had a fit during the night, in which she appeared to have lost the power over her limbs, and the legs were somewhat contracted; the hands were placed over the occiput, where she appeared to suffer pain. She is now rather confused; pupils dilated, though obedient to light. There is some involuntary movement principally confined to the left side. Bowels well opened yesterday; pulse small and soft.

Radatur caput lotio frigida constanter applicand : zinci. sulph. gr. iv. ter die.

March 16. Had another fit this morning, but much less severe than before. Pulse quick; pain in the head not increased.

22. Has had no return of the fits, and appears much improved. The involuntary twitchings are comparatively slight. She is more collected and can articulate clearly. Bowels open; pulse quiet; still deaf; her strength is so increased that this morning she was able to walk twelve or fourteen yards.

℞. Infus rosæ. cum quin. sulph. gr. ii. ter die. Pil rhei. comp. gr. x. p. r. n.

She remained in the hospital till April 21, when she was presented quite well. During this time she gradually regained her strength, losing all symptoms of chorea. The general health was confirmed; her appetite returned; the catamenia appeared though scantily, and her countenance assumed its natural aspect. Her intellect is still somewhat impaired, but the head is free from the occipital pain, and there is no symptom of structural change."

This requires no comment. It attests the extreme severity of the cerebral affection, and its symptoms are so precisely identical with those of organic disease as fully to demonstrate the impossibility of making a certain diagnosis, while, at the same time, it teaches us to be careful of our prognosis.

All the organic affections of the womb are given at length, various modes of treatment fully discussed, and all the subjects admirably illustrated by examples. We have not the space that would be required, even to name all these diseases, therefore we shall not do them the injustice of attempting an analysis.

Occlusion of the uterus, or total absence of an os tincæ, is an accident of so much interest to obstetricians, that we cannot resist the inclination to make some selections from the author's treatise on the subject.

"Happily, entire closure, and such extreme rigidity of the os as to preclude the birth of a child, if help be not afforded, without more or less extensive laceration, are rare. Still two such examples—the one of closure—the other of rigidity—where incision was successfully performed have fallen under my own observation within a short time; and I have already alluded to not a few occurring in the practice of others."

Dr. Ashwell thinks that when the uterus is imperforate, or when the os, though existing, is so rigid as to resist, after hours of strong uterine action, the dilating process, incision is the safest remedy, far preferable, he says, to protracted dilatation of the os by the finger; or, on the principle of non-interference, to leaving the case to the natural efforts.

"It is well known," [says our author,] "that normally, this orifice is sometimes very small; at others, instead of a transverse chink—its most usual form—there is merely a diminutive circular aperture. In either of these conditions of the orifice, complete obliteration may easily be produced by an amount of local inflammation following conception, which would not seriously interfere either with the pregnancy or the health of the individual. It is important, however, to bear in mind, that such closure may not be attended by any other disease of the parts; the adhesion may be firm and complete; but there may be no scirrhous induration, no distinct nodule of hard substance; the neck of the uterus will be forced down by the pains; and the sensation imparted to the finger, on examination during labor, will be quite natural, excepting only that no aperture will be found."

Again, he says:

"It will be urged as extremely improbable that there should be no os uteri; that there is one, perhaps, but that owing to obliquity, it is very high up posteriorly; and that being thus unnaturally situated, twenty-four, thirty-six, or forty-eight hours, or even a longer period, will be required for its development and dilatation."

Dr. Ashwell very justly attributes many of the recorded fatal cases to an unwarranted delay in performing the operation, and truly states, that every hour of urgent uterine action tends to rectify obliquity, if such be the cause of an undiscovered os uteri. If the pains, he says, "are really powerful and protracted, for ten or twelve hours, the os uteri being still undiscovered, it may fairly be assumed that it is wanting."

The writer, of course, admits that in some cases bleeding and tartar emetic will produce the desired relaxation, but he contends that when we are certain that occlusion exists, and the fact of its existence is sometimes beyond the possibility of doubt, to delay the operation will only bring on exhaustion and add considerably to the chances of an unfavorable result. From the foregoing it must not be supposed that it is advised to incise the lower segment of the womb without great caution and a perfect conviction of its necessity; on the contrary, the writer insists upon careful investigation, and likewise on the propriety of previous consultation; laceration will take place, he says, to a certain extent, but seldom beyond the lower segment of the organ included within the reflection of the vaginal mucous membrane; whereas, if the case be left to nature, violent contractions must lead to rupture, involving, in all probability, the cavity of the peritoneum, a fatal accident, or the suffering and exhausted woman will sink from inflammation and gangrene.

The work, of course, includes a long article on ovarian diseases; limited space obliges us to be brief, but the subject has of late acquired a refreshed interest by the endeavors of certain European surgeons to legitimatize the frightful operation of excising these organs when in a morbid condition. We have neither the time nor the inclination at present to undertake a discussion on this unpleasant subject, but by a few quotations, will merely show our author's opinion and feelings respecting the operation.

Thus, speaking of dropsy, he says:

"If the operation is to come into established practice, of which I have the strongest doubt, it must be confined to examples of the malady where tapping has already been so often employed as to preclude, from experience of similar cases, any idea that it can be dispensed with; and where we are confident that great suffering must lead to early death. Perhaps this may be regarded as too limited a view of the value of extirpation, but it is, I think, the correct one."

He further adds:

"The extirpation, we are assured by the operators themselves, in a fit case, is far from difficult—would it were more so, for then it would not be so readily undertaken. If it required as much surgical knowledge and skill to make these large and brilliant abdominal incisions, as to tie the subclavian artery, or to perform a trying operation of lithotomy, the lives of many women would have been already spared, and fewer would be sacrificed for the future."

We are glad to find that the opinion of this most respectable authority so perfectly coincides with that of the profession in this country.

Displacement of the uterus, and likewise diseases of the external organs of generation, are treated in the same thorough and satisfactory manner which distinguishes the author's writing.

We must here stop; it has not been our purpose to give an analysis of the very varied contents of the book, which touches more or less fully on everything interesting connected with the subject. This within our scanty limits would be impossible. If the few specimens given shall induce the reader to examine the work for himself, (and as a reference it is invaluable,) we can assure him that he will not regret it.

---

*The Pathology and Treatment of Leucorrhœa.* By TYLER SMITH, M.D., &c. Philada., Blanchard & Lea, 1855.

The obscurity and confusion which encumbered the pathology of Leucorrhœa, one of the consequences of which was a very frequent and often unnecessary resort to the use of the speculum by some members of the profession, and its almost entire repudiation by others, induced our author, whose previous researches well fitted him for such a task, to make a thorough microscopical examination of the discharges attending the various diseases of the female sexual organs, as well as of the parts involved in their production. The result of his labors is before us in a work which we can safely commend to the profession as one of sterling value, and which cannot fail to prove both interesting and instructive to every one who desires to understand the true pathology of the various diseases of the womb and its appendages.

The first chapter, after stating the author's reasons for adhering to the term "leucorrhœa," treats of the minute anatomy of the vagina, of the os uteri and the vaginal portion of the os and cervix uteri. The tesselated layer of epithelium, and more particularly the villi beneath the basement membrane, are most accurately described. The very beautiful plates which illustrate the text, for the drawings of which the author expresses his obligations to Dr. Hassall, are of great use in enabling the reader to comprehend his verbal descriptions. The following remarks, containing the author's views upon the functions of the villi in the different situations in which they present themselves, are interesting.

"It becomes a question of much interest to decide what are really the functions performed by the villi of the vagina and the os and cervix uteri. In the lower part of the vagina, the papillæ are no doubt sensitive in function, and deserve the term applied to them by Dr. F. Kilian; but in the upper part of the vagina, the mucous surface is, in the healthy condition, possessed of little sensibility either to pleasure or pain; yet the papillæ or villi are very abundant. The villi are present in very great abundance upon the os uteri and the external portion of the cervix; though the os uteri is, in the majority of cases, insensible to the touch. It is only when the os or cervix uteri is distended by instruments, or when the cervical canal is inflamed or constricted, that pain is caused in ordinary cases. The villi are largest and more highly developed within the os uteri, where sensation is more blunted than in any part of the vagina or os uteri. For these reasons, I am inclined to believe that the villi of the os and cervix uteri, particularly the villi of the cervical canal, are little concerned in sensation. From the liberal supply of blood possessed by the villi, I suspect they are concerned in the secretion of the fluid plasma which the external portion of the os and cervix and the upper part of the vagina pour out, and which forms the vehicle in which the epithelial *débris* is suspended; or they may be intended for the formation of the thick layer of epithelium covering these parts, and which is in constant process of renewal and disintegration."

The second chapter treats of the glandular structures of the canal of the cervix uteri. Great importance is attached by the author to the follicular condition of the canal which, from the peculiar disposal of its mucous membrane, affords a very large extent of glandular surface for the purposes of secretion. "The villi found in the upper portion of the cervix are covered by dentated epithelium, just as is the case with the villi of the lowest part of the cervix." The epithelium of the os uteri and external portion of the cervix is, on the contrary, like that of the vagina, constantly squamous; the transition from squamous to dentated epithelium, it is stated, occurs just at the margin of the os uteri.

In the third chapter the healthy secretions of the vagina and of the os and cervix uteri are thoroughly described. The mucus of the ostium vaginæ, secreted by the glands of Duverney, like all the other vaginal secretions, are acid in their reaction. During sexual excitement a profuse emission of this fluid takes place in some women. In the absence of excitement the secretion is inconsiderable. The importance of these glands upon the production of leucorrhœa, has, Dr. Smith thinks, been much over-estimated.

The vaginal mucus is a milky fluid, secreted in quantities sufficient only to lubricate the mucous surface; after remaining upon the vaginal surface a short time it becomes curdled, owing to its acid nature. Spermatozoa preserve their vitality for some time in this normal acid mucus, but are soon destroyed when the acid is in excess from any cause. The secretion from the labia uteri, on the contrary, is always alkaline. These different chemical conditions are considered to be of great importance in explaining many circumstances connected with the discharges in leucorrhœa.

In the unimpregnated state, the canal of the cervix is full of its peculiar secretion; this is washed away, at every period, by the menstrual fluid, to form again when the catamenia have ceased. It is less thick and viscid when first secreted, than it afterwards becomes. The mucous follicles become comparatively inactive after they have thrown off a sufficient quantity of mucus to fill the canal, until the next menstrual flow removes it, when they again become active. "The function of the cervix is, therefore, in a certain sense, like that of the fundus, periodical;" and this periodicity is seen in the diseased conditions of the cervix and its secretions. The mucus secreted consists of mucus corpuscles entangled in a very tenacious transparent plasma. Its use is two-fold; it defends the cavity of the uterus from external agencies, and affords a medium for the passage of the spermatozoa into the uterine cavity.

During pregnancy the plug of viscid mucus continues until the commencement of labor. The chief changes occurring in it are due to the alterations taking place in the cervix; it is firmer than the mucus filling the cervix in the unimpregnated state, particularly at its lowest part, where it is opaque and white. In the upper part of the cervix it is clear and transparent, and of an alkaline reaction. The author thinks that the opaque and white mucous secretion rarely exists except during pregnancy, and gives several cases where its presence was of service in confirming an otherwise doubtful diagnosis. During lactation there is a marked tendency to increased secretion of mucus from the glands of the cervical canal, which sometimes lays the foundation of chronic leucorrhœa.

The fourth chapter contains a full description of the two principal forms of leucorrhœa; the mucous variety, consisting chiefly of mucus-corpuscles and plasma, and secreted by the follicles of the cervical canal; and the epithelial variety, secreted by the vaginal portion of the os and cervix, and consisting of scaly epithelium and its debris. Our space not allowing us to make such extracts from this chapter as we could wish, we must refer our readers to the work itself for information upon the subject.

The fifth chapter on the sequelæ of leucorrhœa is an exceedingly interesting one. The author believes that in the majority of cases in which disease of the os and cervix is present, cervical leucorrhœa is the essential part of the disorder, and that the diseased conditions of the lower segment of the uterus are frequently secondary affections, the result of the augmented secretion. "Every thing," he remarks "has been made to bend to the hypothesis which sets forth inflammation as the keystone of uterine disorder. Laborious disquisitions have been entered into respecting the vascularity of the os and cervix, the existence or non-existence of a mucous membrane in this situation, and the absence or presence of cellular tissue in the substance of the cervix uteri, as conditions which have been supposed necessary to the establishment of the theory of inflammation. I believe the truth, however, to be, that this part of the generative system is the seat of frequent disorder, chiefly from the circumstance that it is the boundary at which cutaneous tissue ends and mucous tissue begins. The vagina must be considered as an internal prolongation of the skin, and it is only at the labia uteri that the true mucous membrane really commences."

The remaining chapters of the work discuss the relations of leucorrhœa to secondary syphilis; to gonorrhœa in the female; urethritis in the male; to the ophthalmia of new-born infants; to disorders of the function of menstruation, and to sterility and abortion. A chapter upon the constitutional and local causes of leucorrhœa, followed by one upon its treatment, completes the work.

We are aware that Dr. Smith's work deserves a much longer and more elaborate notice than our limits have obliged us to give of it. Even the inadequate sketch we have attempted, however, must show what an amount of light his researches have thrown upon the important and much-vexed subject of leucorrhœa. We regard his book as one of the most excellent that has been presented for some time to the medical public.

The publishers have issued the work in most praiseworthy style, the paper, print and plates, which latter are quite numerous, being all of them unexceptionable.

---

*Observations on Wounds of the Heart, and their relations to Forensic Medicine.* By SAMUEL PURPLE, M.D., &c. New York.

*On the Chemical Analysis of the Tennessee Collection of Urinary Calculi.* By E. B. HASKINS, M.D. Clarksville.

We can heartily commend both of the above pamphlets to our readers' attention as papers of real and permanent value. Dr. Purple's observations show, contrary to the general opinion held upon the subject, that even severe wounds of the heart are not necessarily fatal, several instances being related where the recipients of such wounds lived for months, and two where they lived for years afterwards. A table of 42 cases, in which the nature of the injury, the primary symptoms and progress, the result and post-mortem appearance, are all recorded, affords the author an opportunity of settling many important questions regarding these wounds. Sudden loss of blood, compression of the heart from accumulation of blood in the pericardium, concussion or shock of the organ and inflammation, are the principal causes of death in these cases. The symptoms vary very little in their character. "On the receipt of injury we have generally hæmorrhage, weakness, syncope, great paleness, great anxiety and oppression, cold sweat, vomiting, with sooner or later, not unfrequently, complete collapse. The breathing is short and gasping, the pulse feeble and intermittent, the heart's action, scarcely, if at all, perceptible. In the course of from one to eight hours, signs of reaction set in ; not unfrequently after vomiting the dyspnœa and cardiac oppression are in a measure relieved ; then palpitations not unfrequently arise, with a peculiar trembling of the organ, with irregular pulse." The diagnosis of such wounds, however, is often illusive. So uncertain is it deemed by the author, that no case of reputed recovery is introduced into his table, where the rational symptoms were alone relied upon.

As regards the treatment, there being less to be feared from the collapse than from the subsequent reaction and fever, a free resort to stimulants should be avoided. To promote the formation of a clot in the wound, adhesive plaster and bandages should be applied to the external aperture, and these should only be removed where there "is reason to believe that the dyspnœa and sense of suffocation are greatly increased by restraining the external hæmorrhage." When reaction sets in, we are to depend upon bleeding and strict antiphlogostic treatment to subdue it. To diminish the heart's action, the veratrum viride is strongly recommended; a moderate mercurialization is also advised as a safeguard against the inflammatory complications which so frequently arise. The necessity of perfect, absolute rest, and for weeks, is forcibly inculcated by reference to cases in which it was neglected or could not be enforced.

The author's remarks upon the prognosis of these wounds and their forensic relations are well considered and judicious; the paper closes with a series of "conclusions," deductions from the facts related. We consider Dr. Purple's monograph a very excellent one, and cheerfully commend it to general attention.

Dr. Haskins' report embraces the results of the chemical analysis of 180 urinary calculi, all but four of which were from human subjects. Contrary to the statements of British and Continental writers, who, all of them, speak of the uric acid calculus as the most common variety, Dr. H. found it extremely rare, while urates were quite common. It would be interesting to know, as the author remarks, to what extent this peculiarity in the urinary deposits exists in the United States, and whether it is confined to the Mississippi valley? We commend the subject to the attention of northern chemists. From facts recorded by him, Dr. H. believes that few calculi owe their origin to constitutional causes, the great majority of them arising from local ones. "It can hardly be doubted," he says, "that the little masses of animal matter, in the form of epithelium debris, fibrinous and perhaps albuminous exudation, and drops of blood, acted as the potential causes of the formation of the concretion in which they were found." The urine may not have been abnormal in its composition or sedimentous in any of these cases for these bodies to become nuclei of deposit, as it is well known that any foreign body in the urinary passages speedily provokes a precipitation, even from healthy urine. The paper concludes as follows: "I wish only to show by the foregoing remarks, that without a nucleus of foreign matter, *no* state of the urine (with the few exceptions mentioned) will likely give origin to a calculus, and that where there is such a nucleus, *any* state of the urine may form one. And, that the facts above recorded point to the kidney and the agents that determine its local pathology, rather than to the urine and those agents that modify its chemical nature, as the chief sources of urinary calculi."

For the industry and perseverance with which he has pursued his investigations, which must have been exceedingly laborious, Dr. Haskins deserves great credit. In fact, his report cannot fail to be regarded, wherever it is read, as a very valuable contribution to science. It was originally published in the Transactions of the Tennessee Medical Society.

